# Supplementary material for: Altruistic punishment does not increase with the severity of norm violations in the field
Source: Nat Commun. 2016 Nov 1;7:13327. doi: 10.1038/ncomms13327 (PMC5097122; doi:10.1038/ncomms13327)
Supplement: Supplementary Information — Supplementary Note 1, Supplementary Methods and Supplementary References [file ncomms13327-s1.pdf]

Supplementary Note 1:

1) A methodological difference between Survey 1 and Survey 2 is that, in Survey 2, respondents were shown both violations and asked to directly compare them. This approach is inspired by Wolfgang *et al.*[1] and allows us to obtain within-subject data on the way that individuals perceive the small and large violation relative to each other. This is particularly important for evaluating the perceived severity of the violation.

2) To control for presentation effects, we conducted Survey 2 in two different versions, A and B. In version A (B), the small (large) violation was shown on the left and listed first in the possible responses to questions 2-5. We collected equal amounts of versions A and B. We find no significant differences between the two versions in the distribution of responses to any of the questions ($p>0.3$, chi-squared tests for each question separately); therefore we present here pooled data for both versions of Survey 2.

3) In Survey 2 we use Wilcoxon signed ranks tests for paired samples in order to test for statistical significance between the two violations in questions 2-5. In particular, we assign a value of 0 to responses indicating no difference between the two violations, and a value of 1 (-1) when the large (small) violation is considered more severe (in question 2), more bothersome (in question 3), deserving of more punishment (in question 4), or deserving of less help (in question 5). Deviations from 0 that go systematically in one direction are indicative of treatment differences. We note that in question 2, question 3 and question 4 almost all observed deviations from 0 are in the direction of the large violation, leading to highly significant treatment differences (Wilcoxon sign-rank test; question 2: $z=6.621$, $p<0.001$, $N=324$; question 3: $z= 9.390$, $p<0.001$, $N=324$; question 4: $z= 5.060$, $p<0.001$, $N=324$). These tests are also reported in the manuscript. We find no significant treatment differences in responses to question 5 (Wilcoxon sign-rank test $z=0.378$, $p=0.71$, $N=324$).

Supplementary Methods

The supplementary methods provide the three surveys: Survey 1 - small violation, Survey 1 - large violation, and Survey 2. Each survey is presented in the original German version with English translation in italics in parentheses. The percentage numbers in the tick boxes refer to the fraction of responses given.

1. Survey 1 - small violation

"Guten Tag. Wir sind ein Forschungsteam aus den Universitäten von Köln, Innsbruck und New York. Wir führen eine Umfrage über den Zustand der Bahnhöfe durch. Hätten Sie bitte zwei Minuten Zeit, um ein paar Fragen zu beantworten bis Ihr Zug angekommen ist?" *("We are a team of researchers from the Universities of Cologne, Innsbruck and New York. In agreement with Deutsche Bahn we are conducting a survey regarding the conditions in train stations. Could you please spare us two minutes of your time and answer a few questions?")*

1. Wie oft fahren Sie üblicherweise mit dem Zug?
(How often do you usually take the train?)

Fünf Tage die Woche oder mehr *(Five days a week or more)*	46.0%	Drei bis vier Tage die Woche *(Three to four days a week)*	22.6%
Einen bis drei Tage die Woche *(One to three days a week)*	7.6%	Weniger als einmal die Woche *(Less than once a week)*	23.8%

2. Stellen Sie sich die folgende auf dem Bild abgebildete Situation vor: Sie bemerken eine Frau, die einen leeren Kaffeebecher auf den Bahnsteig wirft. Würde Sie das stören?
(Imagine the following situation shown on this picture: you notice a woman who throws an empty coffee cup on the train platform. Would this bother you?)

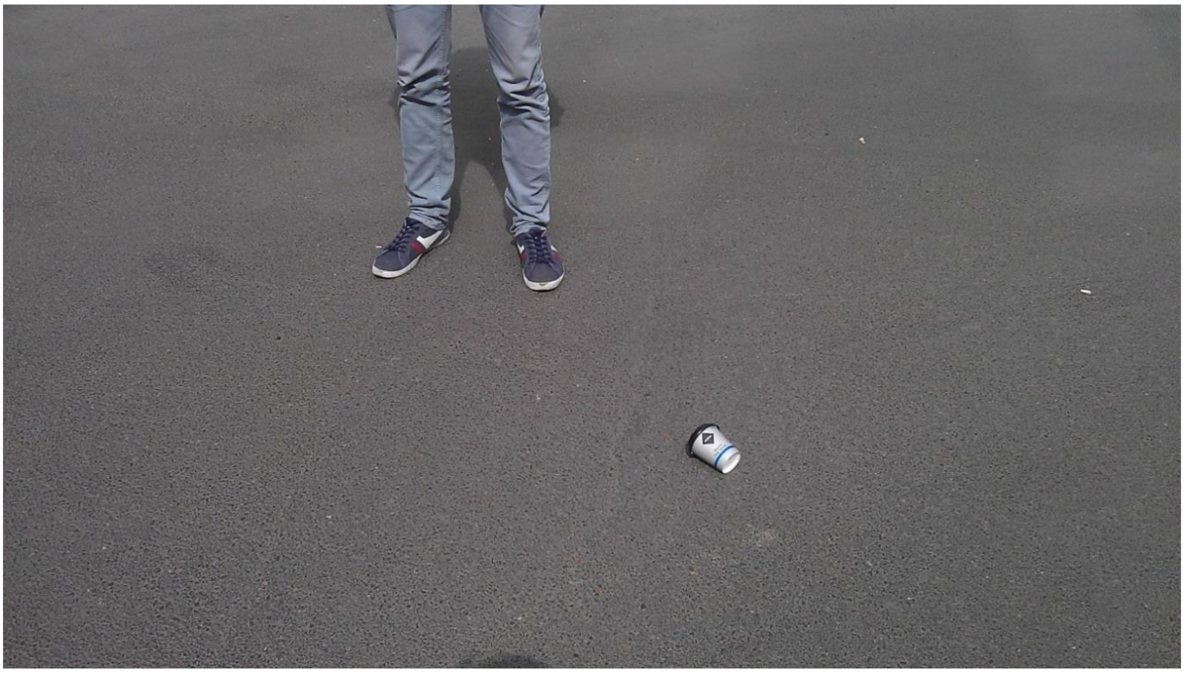

Nein<br>*(No)*	6.4%
Ja – ein bisschen<br>*(Yes – a little)*	24.2%
Ja – ziemlich<br>*(Yes – quite a lot)*	36.1%
Ja – sehr<br>*(Yes – a lot)*	33.3%

3. [Wenn die Antwort auf (2) JA ist] Würden Sie die Frau zurechtweisen?
([If the answer to (2) is YES] Would you reproach the woman?)

Nein<br>*(No)*	54.9%
Ja<br>*(Yes)*	45.1%

4. [Wenn die Antwort auf (3) NEIN ist] Aus welchem Grund/welchen Gründen?
([If the answer to (3) is NO] For which reason(s)?)

Weil das zu Streit führen könnte<br>*(Because that could lead to a dispute)*	53.7%
Weil niemand Leute zurechtweist, die leere Kaffeebecher wegwerfen<br>*(Because no one reproaches someone who throws away their empty coffee cup)*	2.8%
Weil es dafür Reinigungskräfte gibt<br>*(Because it is the job of cleaning personnel to pick up litter)*	2.8%
Weil man Anderen nicht vorschreiben sollte, wie sie sich verhalten<br>*(Because no one should tell others how to behave)*	37.9%
Anderes<br>*(Other)*________________________________	2.8%

5. Wie viele Zugpassagiere wissen Ihrer Meinung nach, dass sie keine leeren Kaffeebecher auf den Bahnsteig werfen dürfen?
(In your opinion, how many train passengers know that they should not throw empty coffee cups on the platform?)

Keiner<br>*(No one)*	0.4%
Wenige<br>*(A few)*	5.6%
Ziemlich viele<br>*(Quite a few)*	6.3%
Die meisten<br>*(Most)*	21.4%
Alle<br>*(Everyone)*	66.3%

6. Jetzt stellen Sie sich vor, die Frau, die den Kaffeebecher weggeworfen hat, lässt dann aus Versehen ihre Bücher genau vor Ihnen auf den Bahnsteig fallen. Würden Sie ihr helfen, die Bücher aufzuheben?

(Now imagine that the woman who threw her coffee cup accidentally drops her books directly in front of you on the platform. Would you help her to pick up the books?)

Ja<br>*(Yes)*	18.3%
Nein<br>*(No)*	78.2%
Ich würde es lieber vermeiden<br>*(I would rather avoid it)*	3.5%

7. Bitte geben Sie uns eine kurze Begründung für Ihre Antwort:

(Please give a brief explanation for your response)

____

____

8. Geschlecht:
(Gender)

Männlich<br>*(Male)*	46.0%
Weiblich<br>*(Female)*	54.0%

9. Alter:
***(Age)* _______**(mean = 42.2 years)

Datum:
(*Date*) ___________

Interviewer:
(*Interviewer*) ___________

2. Survey 1 - large violation

"Guten Tag. Wir sind ein Forschungsteam aus den Universitäten von Köln, Innsbruck und New York. Wir führen eine Umfrage über den Zustand der Bahnhöfe durch. Hätten Sie bitte zwei Minuten Zeit, um ein paar Fragen zu beantworten bis Ihr Zug angekommen ist?"
("We are a team of researchers from the Universities of Cologne, Innsbruck and New York. In agreement with Deutsche Bahn we are conducting a survey regarding the conditions in train stations. Could you please spare us two minutes of your time and answer a few questions?")

1. Wie oft fahren Sie üblicherweise mit dem Zug?
How often do you *usually* take the train?

Fünf Tage die Woche oder mehr<br>*(Five days a week or more)*	47.7%	Drei bis vier Tage die Woche<br>*(Three to four days a week)*	20.5%
Einen bis drei Tage die Woche<br>*(One to three days a week)*	8.9%	Weniger als einmal die Woche<br>*(Less than once a week)*	22.9%

2. Stellen Sie sich die folgende auf dem Bild abgebildete Situation vor: Sie bemerken eine Frau, die nach dem Essen ihre Papiertüte auf den Bahnsteig wirft, aus der benutzte Papierservietten und ein Kaffeebecher herausfallen.
(Imagine the following situation shown on this picture: you notice a woman who throws a large paper bag on the train platform, with used napkins and an empty coffee cup falling out. Would this bother you?)

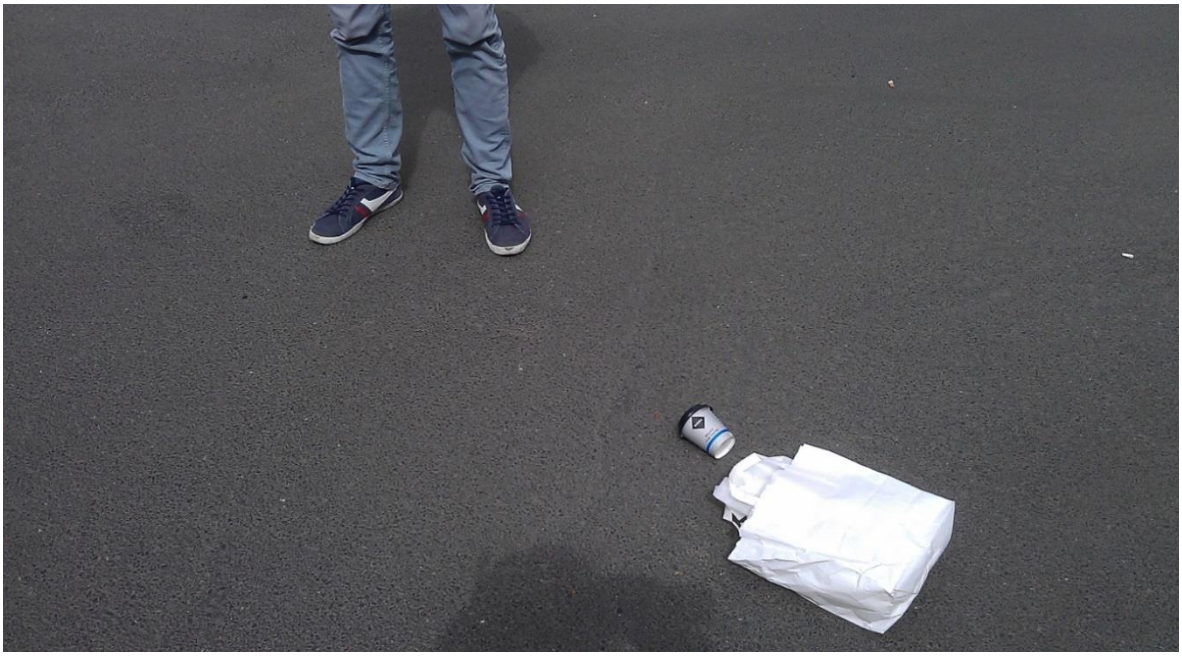

Nein<br>*(No)*	5.0%
Ja – ein bisschen<br>*(Yes – a little)*	18.2%
Ja – ziemlich<br>*(Yes – quite a lot)*	29.1%
Ja – sehr<br>*(Yes – a lot)*	47.7%

3. [Wenn die Antwort auf (2) JA ist] Würden Sie die Frau zurechtweisen?
([If the answer to (2) is YES] Would you reproach the woman?)

Nein<br>*(No)*	50.4%
Ja<br>*(Yes)*	49.6%

4. [Wenn die Antwort auf (3) NEIN ist] Aus welchem Grund/welchen Gründen?
([If the answer to (3) is NO] For which reason(s)?)

Weil das zu Streit führen könnte<br>*(Because that could lead to a dispute)*	67.0%
Weil niemand Leute zurechtweist, die leere Kaffeebecher wegwerfen<br>*(Because no one reproaches someone who throws away their empty coffee cup)*	4.9%
Weil es dafür Reinigungskräfte gibt	1.9%

(Because it is the job of cleaning personnel to pick up litter)	
Weil man Anderen nicht vorschreiben sollte, wie sie sich verhalten *(Because no one should tell others how to behave)*	25.2%
Anderes *(Other)* ______________________________	1.0%

5. Wie viele Zugpassagiere wissen Ihrer Meinung nach, dass sie keine leeren Kaffeebecher auf den Bahnsteig werfen dürfen?
(In your opinion, how many train passengers know that they should not throw empty coffee cups on the platform?)

Keiner *(No one)*	0.4%
Wenige *(A few)*	3.5%
Ziemlich viele *(Quite a few)*	8.1%
Die meisten *(Most)*	21.3%
Alle *(Everyone)*	66.7%

6. Jetzt stellen Sie sich vor, die Frau, die ihre Papiertüte weggeworfen hat, lässt dann aus Versehen ihre Bücher genau vor Ihnen auf den Bahnsteig fallen. Würden Sie ihr helfen, die Bücher aufzuheben?
(Now imagine that the woman who threw her paper bag accidentally drops her books directly in front of you on the platform. Would you help her to pick up the books?)

Ja *(Yes)*	16.3%
Nein *(No)*	78.3%
Ich würde es lieber vermeiden *(I would rather avoid it)*	5.4%

7. Bitte geben Sie uns eine kurze Begründung für Ihre Antwort:

(Please give a brief explanation for your response)

____

____

8. Geschlecht:
(Gender)

Männlich *(Male)*	46.1%
Weiblich *(Female)*	53.9%

9. Alter:
(Age) **_______**(mean = 41.3 years)

Datum:
(*Date*) **___________**

Interviewer:
(*Interviewer*) **___________**

3. Survey 2

"Guten Tag. Wir sind ein Forschungsteam aus den Universitäten von Köln, Innsbruck und New York. Wir führen eine Umfrage über den Zustand der Bahnhöfe durch. Hätten Sie bitte zwei Minuten Zeit, um ein paar Fragen zu beantworten bis Ihr Zug angekommen ist?"
("We are a team of researchers from the Universities of Cologne, Innsbruck and New York. In agreement with Deutsche Bahn we are conducting a survey regarding the conditions in train stations. Could you please spare us two minutes of your time and answer a few questions?")

1. Wie oft fahren Sie üblicherweise mit dem Zug?
(How often do you usually take the train?)

Fünf Tage die Woche oder mehr *(Five days a week or more)*	19.2%	Drei bis vier Tage die Woche *(Three to four days a week)*	7.7%
Einen bis drei Tage die Woche *(One to three days a week)*	14.5%	Weniger als einmal die Woche *(Less than once a week)*	58.6%

2. Stellen Sie sich bitte die auf den folgenden zwei Bildern abgebildeten Situationen vor:
(Imagine the following situations shown on the two pictures below)

Sie bemerken eine Frau, die eine **große Papiertüte** auf den Bahnsteig wirft, aus der benutze Papierservietten und ein Kaffeebecher herausfallen
*(You notice a woman who throws **a large paper bag** on the train platform, with used napkins and an empty coffee cup falling out)*

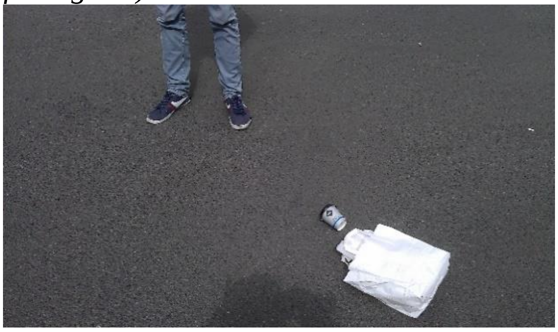

oder
(or)

Sie bemerken eine Frau, die einen **leeren Kaffeebecher** auf den Bahnsteig wirft
*(You notice a woman who throws **an empty coffee cup** on the train platform)*

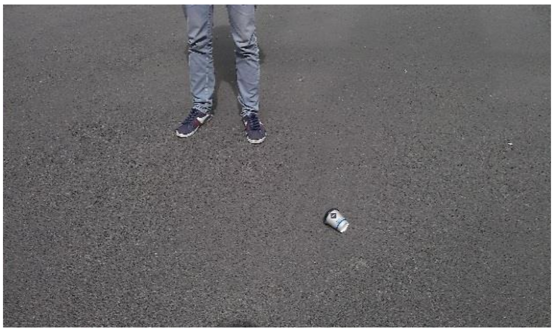

2. Welches Verhalten ist Ihrer Meinung nach schlimmer?
(Which behavior is more severe in your opinion?)

19.1%	Eine große Papiertüte auf den Bahnsteig zu werfen ist schlimmer *(Throwing a large paper bag on the train platform is more severe)*
2.2%	Einen leeren Kaffeebecher auf den Bahnsteig zu werfen ist schlimmer *(Throwing an empty coffee cup on the train platform is more severe)*
78.7%	Keines der zwei Verhalten ist schlimmer als das andere *(None of the two behaviors is more severe than the other)*

3. Welches Verhalten würde Sie mehr stören?
(Which behavior would bother you more?)

33.9%	Eine große Papiertüte auf den Bahnsteig zu werfen würde mich mehr stören *(Throwing a large paper bag on the train platform would bother me more)*
2.5%	Einen leeren Kaffeebecher auf den Bahnsteig zu werfen würde mich mehr stören *(Throwing an empty coffee cup on the train platform would bother me more)*
63.6%	Keines der zwei Verhalten würde mich mehr stören als das andere *(None of the two behaviors would bother me more than the other)*

4. Welches Verhalten sollte man Ihrer Meinung nach stärker zurechtweisen?
(Which behavior should be reprimanded more strongly in your opinion?)

11.1%	Eine große Papiertüte auf den Bahnsteig zu werfen sollte man stärker zurechtweisen *(Throwing a large paper bag on the train platform should be reprimanded more strongly)*
1.2%	Einen leeren Kaffeebecher auf den Bahnsteig zu werfen sollte man stärker zurechtweisen *(Throwing an empty coffee cup on the train platform should be reprimanded more strongly)*
87.7%	Keines der zwei Verhalten sollte man stärker zurechtweisen als das andere *(None of the two behaviors should be reprimanded stronger than the other)*

5. Stellen Sie sich vor, der Frau passiert ein Missgeschick nachdem sie den Bahnsteig verschmutzt hat: ihr fallen Zeitschriften aus ihrer Tasche. Würden Sie ihr beim Aufheben helfen?
(Now imagine that the woman has a mishap after she pollutes the platform: books fall out of her bag. Would you help her to pick up the books?)

1.2%	Ich würde eher einer Frau helfen, die eine große Papiertüte auf den Bahnsteig geworfen hat *(I would rather help the woman who threw a large paper bag on the train platform)*
0.9%	Ich würde eher einer Frau helfen, die einen leeren Kaffeebecher auf den Bahnsteig geworfen hat *(I would rather help the woman who threw an empty coffee cup on the train platform)*
20.1%	Ich würde in keinem der beiden Fälle helfen *(I would not help in any of the two cases)*
77.8%	Ich würde in beiden Fällen gleich viel helfen

☐ *(I would help equally in both cases)*

Bitte geben Sie eine kurze Begründung Ihrer Antwort:

(Please give a brief explanation for your response)

____

____

6. Geschlecht:
(Gender)

Männlich<br>*(Male)*	50.3%
Weiblich<br>*(Female)*	49.7%

7. Alter:
(Age) _______(mean = 46.6 years)

Datum:
(*Date*) ____________

Interviewer:
(*Interviewer*) ____________

Supplementary References

1. M. E. Wolfgang, R. M. Figlio, P. E. Tracy, S. I. Singer. The National Survey of Crime Severity. Washington, DC: U.S. Department of Justice, Bureau of Justice Statistics (1985).
